# The Role of Targeted Temperature Management in Adult Patients Resuscitated from Nonshockable Cardiac Arrests: An Updated Systematic Review and Meta-Analysis

**DOI:** 10.1155/2016/2350974

**Published:** 2016-10-25

**Authors:** Lijuan Song, Liang Wei, Lei Zhang, Yubao Lu, Kaifa Wang, Yongqin Li

**Affiliations:** ^1^School of Biomedical Engineering, Third Military Medical University, Chongqing 400038, China; ^2^Emergency Department, Southwest Hospital, Third Military Medical University, Chongqing 400038, China; ^3^Emergency Department, Xinqiao Hospital, Third Military Medical University, Chongqing 400037, China

## Abstract

Routine targeted temperature management is recommended for comatose adult patients with return of spontaneous circulation after cardiac arrest. However, the role of targeted temperature management in patients resuscitated from nonshockable cardiac arrests remains uncertain. We conducted an updated systematic review and meta-analysis to evaluate the effects of targeted temperature management in this population. Medline, EMBASE, and Cochrane databases were systematically reviewed for studies published between January 2005 and March 2016, in which targeted temperature management was compared with standard care or normothermia for adult patients resuscitated from nonshockable cardiac arrests. A total of 25 trials that included 5715 patients were identified from 10985 relevant papers. Pooled data showed that targeted temperature management not only associated with improved short-term survival (RR = 1.42, 95% CI: 1.28–1.57) and neurological function (RR = 1.63, 95% CI: 1.39–1.91) but also associated with improved long-term survival (RR = 1.64, 95% CI: 1.27–2.12) and neurological recovery (RR = 1.42, 95% CI: 1.07–1.90) in observational cohort studies. However, more frequent infectious complications were reported in hypothermia-treated patients (RR = 1.46, 95% CI: 1.26–1.70) and the quality of the evidence ranged from moderate to very low.

## 1. Introduction

With an incidence ranging from 35 to 125 cases per 100,000 people, out-of-hospital cardiac arrest (OHCA) remains a major public health problem all over the world [1]. Despite advances in resuscitation science and standardization of advanced life support, the less than 10% overall survival rate remains unsatisfactory [2]. The presenting ECG rhythm in cardiac arrest patients may be either a shockable rhythm (ventricular fibrillation (VF) or pulseless ventricular tachycardia (VT)) or a nonshockable one (pulseless electrical activity (PEA) or asystole), depending on the etiology and downtime [3]. Epidemiologic trends suggest that the incidence of OHCA with initial nonshockable rhythms has been growing during the last two decades [4], and the prognosis of such OHCAs is poor, with a survival rate less than 5% [5].

Failure of neurological recovery is the main cause of morbidity and mortality after spontaneous circulation has been restored [6]. Earlier randomized trials demonstrated that targeted temperature management (TTM) improves cerebral recovery [7, 8]. When the body temperature was maintained between 32°C and 36°C for 12 to 24 hrs, survival and neurological outcomes were significantly improved compared to the instances in which TTM was not performed [9]. Accordingly, routine TTM is strongly recommended for comatose adult patients with return of spontaneous circulation (ROSC) after cardiac arrest [10]. However, the level of evidence for the recommendation is different regarding the presenting initial rhythms. For patients with shockable rhythms, the evidence is based on randomized controlled trials (RCTs). For patients with nonshockable rhythms, the benefits of TTM remain conflicting and the recommendation is based on consensus of expert opinion [10].

Because OHCA patients with nonshockable initial rhythms have a lower survival rate than patients with shockable rhythms, RCTs in these patients will require extremely large sample sizes to test the efficacy of TTM. A prior meta-analysis published by Kim et al. [11] with pooled data through March 2010 suggested that TTM was associated with improved short-term survival in adults patients resuscitated from nonshockable OHCAs but was limited by the small sample size (14 trials, 1382 patients). After that, several large observational cohort studies (OCSs) have focused on the role of TTM in this population, reporting conflicting results [12–17]. In the current study, we aimed to conduct an updated and comprehensive systematic review and meta-analysis on the role of TTM in adult cardiac arrest patients presenting with nonshockable initial rhythms. The results of this systematic review may represent an opportunity to provide valuable information for future clinical trials [18].

## 2. Methods

### 2.1. Data Search

PRISMA guideline for randomized trials and MOOSE guideline for observational studies were followed for this review [19, 20]. Medline, EMBASE, and Cochrane databases were systematically reviewed for studies published between January 2005 and March 2016, in which TTM was compared with standard care or normothermia for adult patients resuscitated from nonshockable cardiac arrests. Keywords of “hypothermia” and “cooling” were used for literature search, filtered with the term “arrest” or “cardiac arrest” or “heart arrest” and “non-shockable” or “asystole” or “PEA” or “non-VF”. The search strategy for electronic databases is listed in Supplemental Table  1 (in Supplementary Material available online at http://dx.doi.org/10.1155/2016/2350974). The electronic search was limited to studies on adult human subjects. References from the studies identified and other relevant review articles were also searched to identify other potentially eligible citations. The protocol (SGX2016WZ02) was waived for ethical approval by the Review Board of Third Military Medical University and the study had not been registered on websites.

### 2.2. Study Selection

Two reviewers (LZ and YL) independently screened the identified studies for eligibility, with discrepancies resolved by consensus. The inclusion criteria were (1) RCT or OCS; (2) studies reporting original data about the outcome (such as short-term survival or neurological outcome to hospital discharge and/or long-term survival or neurological outcome); (3) comparative studies (randomized or observational) between TTM (maintaining a targeted temperature between 32°C and 36°C) and standard of care or normothermia (maintaining body temperature > 36°C with or without temperature intervention). Exclusion criteria were age less than 18 years and patients who received combined therapies, such as prehospital and hospital cooling, TTM, and percutaneous coronary intervention.

### 2.3. Study Outcome Definition

The primary outcomes of interest were short-term survival or neurological outcome. Favorable short-term outcomes were defined as survival or good neurologic recovery at discharge from the hospital, or until 30 days after cardiac arrest. The secondary outcomes were long-term survival or neurological outcome. Favorable long-term outcomes were defined as being alive 6 months after the event or with good neurologic recovery [21]. Good neurological recovery was defined as a Cerebral Performance Category (CPC) score of 1 or 2. If studies reported only good neurological recovery, we considered this outcome to be CPC 1 or CPC 2 [11].

### 2.4. Adverse Events

The adverse events were reported as described by the study authors [9].

### 2.5. Assessment of the Risk of Bias and Quality of Evidence in the Included Studies

To assess the internal validity of identified RCTs, we assessed allocation sequence generation, allocation concealment, blinding of outcome assessment, exclusion of randomized participants from the analysis, comparability of groups, loss to follow-up, and other potential sources of bias using the methodology recommended by the Cochrane Collaboration [22]. As blinding of the intervention with TTM is inherently difficult or impossible, we considered blinding adequate if the outcome assessors had been blinded to the allocation group.

The quality of OCSs was assessed using the Newcastle-Ottawa Scale (NOS) [23]. The NOS consists of 3 categories of quality (selection, comparability, and outcome) and assigns a maximum of 4 stars for selection, a maximum of 2 stars for comparability, and a maximum of 3 stars for outcome.

The evidence was summarized by applying Grading of Recommendations Assessment, Development, and Evaluation (GRADE) levels of high, moderate, low, and very low based on the assessment of the design, limitation, inconsistency, indirectness, imprecision, and possible publication bias of the included studies using the GRADE Pro version 3.6 software [24].

### 2.6. Data Analysis

The meta-analysis of the included studies was performed using Review Manager 5.3 software. Individual and pooled statistics were calculated as risk ratio (RR) with 95% confidence interval (CI). The heterogeneity among the studies was quantified by chi-square test. Values of *I* ^2^ > 50% and *P* < 0.1 indicated significant heterogeneity [25]. When there was no heterogeneity among the studies, pooled effect estimate was assessed using a fixed-effects model. Otherwise, a random-effects model was used.

## 3. Results

### 3.1. Literature Search Results and Characteristics of Included Studies

**Figure 1:**
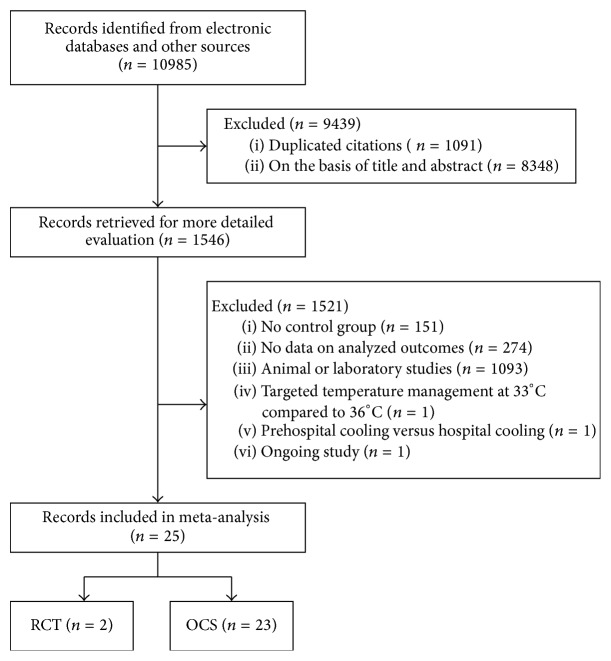
Flow diagram of the literature search.

The flow diagram of literature search is detailed in Figure 1. A total of 25 studies, including 2 RCTs and 23 OCSs, were identified from 10985 relevant papers. External, internal, or combined cooling techniques were used to induce hypothermia and target core temperature was 32–34°C. The duration of TTM was between 18 and 25 hours while normothermia was regained by either active or passive rewarming.

The characteristics of the eligible studies are shown in Table 1. The 2 RCTs were small, single center trials including 44 OHCA patients [26, 27]. Both RCT trials reported 6-month survival and only 1 study reported 6-month neurological recovery. Among the 23 OCSs including 5671 patients, 17 studies reported survival to hospital discharge [14–17, 29–35, 37, 38, 41–44], 2 studies reported 30-day survival [28, 40], 17 studies assessed neurological function at hospital discharge [13, 14, 16, 17, 28–31, 33, 34, 36–39, 41–43], and 1 study reported 14-day neurological outcome [39]. Additionally, 3 OCSs reported 6-month survival [12, 15, 35], and 2 OCSs reported 6-month neurological recovery [12, 15].

### 3.2. The Effects of TTM on Short-Term Outcome

**Figure 2:**
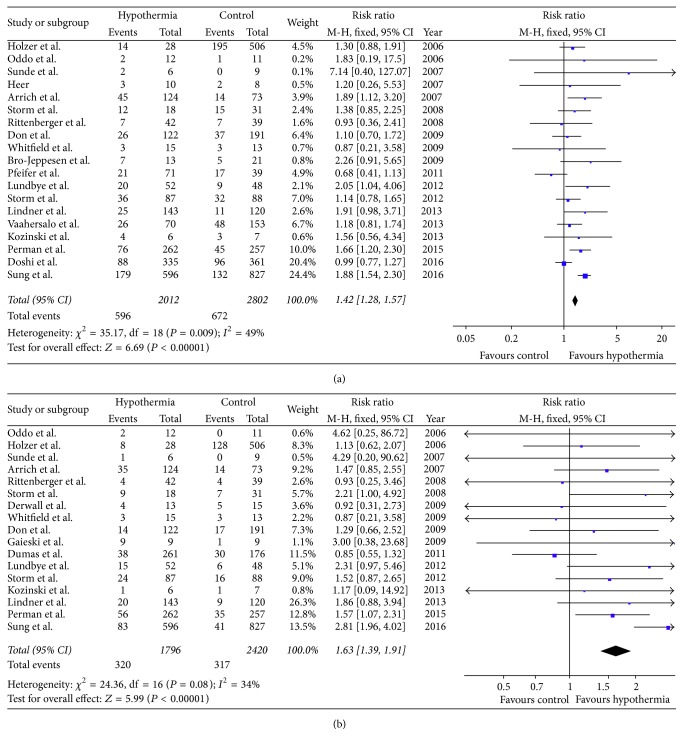
The effects of targeted temperature management on short-term survival (a) and neurological outcome (b) in observational cohort studies.

Nineteen studies involving 4814 patients reported short-term survival in OCSs. Test of heterogeneity was *I* ^2^ = 49% and *P* = 0.009. The pooled result showed that TTM produced significant improvements in survival (RR = 1.42, 95% CI: 1.28–1.57, and *P* < 0.01) using fixed-effects model (Figure 2(a)). Sensitivity analysis was performed as the *I* ^2^ value was very close to the predefined significance level. The relative risk of short-term survival still favored TTM (RR = 1.35, 95% CI: 1.14–1.60, and *P* < 0.01) using random-effects model. When trials with small sample size were excluded, conclusions remain unchanged.

Seventeen studies involving 4216 patients reported short-term neurological outcome in OCSs. Test of heterogeneity was *I* ^2^ = 34% and *P* = 0.08. Meta-analysis indicated that hypothermic group had better neurological recovery than normothermic control (RR = 1.63, 95% CI: 1.39–1.91, and *P* < 0.01) using fixed-effects model (Figure 2(b)).

### 3.3. The Effects of TTM on Long-Term Outcome

**Figure 3:**
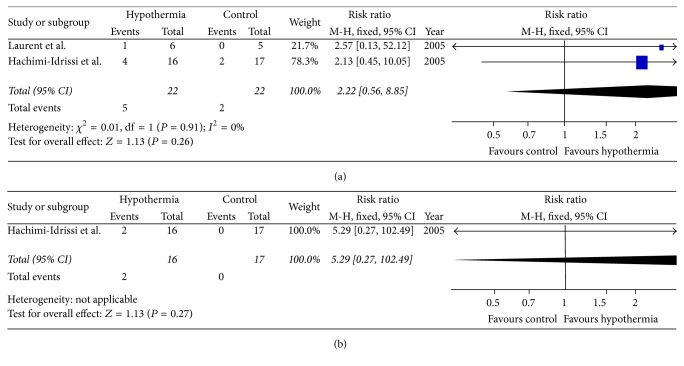
The effects of targeted temperature management on long-term survival (a) and neurological outcome (b) in randomized controlled trials.

Two studies reported long-term survival in RCTs. Test of heterogeneity was *I* ^2^ = 0% and *P* = 0.91. No significant difference was observed in 6-month survival (RR = 2.22, 95% CI: 0.56–8.85, and *P* = 0.26) between hypothermic group and normothermic control using fixed -effects model (Figure 3(a)).

One study involving 33 patients reported long-term neurological recovery in RCTs. The result showed that TTM was not associated with good neurological outcome (RR = 5.29, 95% CI: 0.27–102.49, and *P* = 0.27) using fixed-effects model (Figure 3(b)).

**Figure 4:**
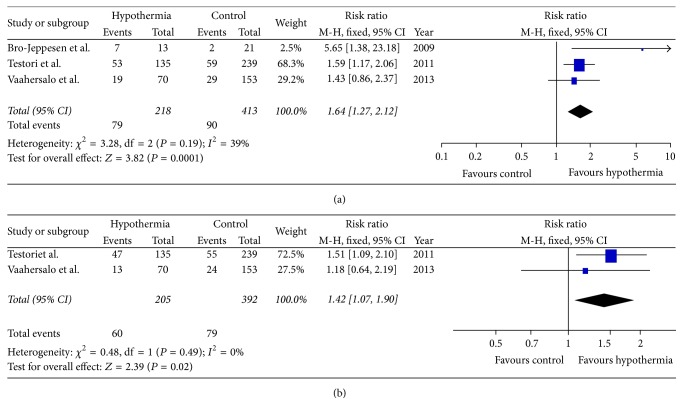
The effects of targeted temperature management on long-term survival (a) and neurological outcome (b) in observational cohort studies.

Three OCSs involving 631 patients reported long-term survival and the pooled data showed that TTM significantly improved 6-month survival (RR = 1.64; 95% CI: 1.27–2.12; *P* < 0.01) using fixed-effects model (Figure 4(a)). Test of heterogeneity was *I* ^2^ = 39% and *P* = 0.19.

Two OCSs involving 597 patients reported long-term neurological recovery. Participants in the hypothermic group were more likely to reach a favorable neurological outcome (RR = 1.42, 95% CI: 1.07–1.90, and *P* = 0.02) using fixed-effects model (Figure 4(b)). Test of heterogeneity was *I* ^2^ = 0% and *P* = 0.49.

### 3.4. Adverse Outcomes

**Figure 5:**
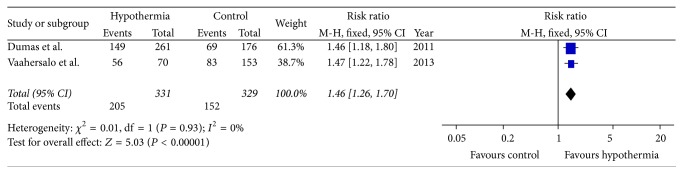
The effects of targeted temperature management on infectious complication in observational cohort studies.

A total of 8 studies [13, 15, 17, 28–31, 43] reported adverse events while only 2 studies [13, 15] compared incidence of complications between TTM and control for patients with presenting nonshockable rhythms. As shown in Figure 5, pooled data showed that infectious complications were more frequent (RR = 1.46, 95% CI: 1.26–1.70, and *P* < 0.01) in hypothermia-treated patients.

### 3.5. Risk of Bias and Quality in Included Studies

The 2 RCTs had substantial risks of bias according to Cochrane methodology (Supplemental Table  2). Sequence generation, allocation concealment, baseline imbalance, and sample size calculations were uncertain in 1 study [26]. Blinding of outcome assessors and baseline imbalance were not reported in the other study [27].

The average NOS score was 7.6 and all of these studies were of high quality (NOS score > 6) (Supplemental Table  3). Among the 17 OCSs in which patients with nonshockable initial rhythms were examined by subgroups, 13 studies (56.5%) did not report comparability of subgroup cohorts [14, 28, 30–36, 38–40, 43] and 2 studies reported significant difference of characteristics between TTM and control [13, 15]. Among the 6 OCSs specifically designed to investigate the effects of hypothermia for nonshockable cardiac arrests, 5 studies (83.3%) reported significant differences in patient demographics and arrest characteristics [12, 16, 41, 42, 44].

Based on the summary of the GRADE methodology (Supplemental Table  4), the accumulated qualities were very low for primary outcome and were low for secondary outcome.

## 4. Discussion

Although TTM has been consistently demonstrated to improve outcomes for patients resuscitated from cardiac arrest with shockable rhythms, its use in subjects with nonshockable ones has produced conflicting results. In a previous meta-analysis, Kim et al. [11] examined the evidence for beneficial effects of TTM in patients who experienced nonshockable cardiac arrests using pooled data from 2 RCTs (involving 22 cases and 22 controls) and 12 OCSs (involving 412 cases and 926 controls) and concluded that TTM was associated with reduced in-hospital mortality (RR = 0.84, 95% CI: 0.78–0.92). In a subsequent review, Sandroni et al. [45] reexamined the effects of TTM for patients who experienced nonshockable cardiac arrests in OCSs (812 cases and 1238 controls) with 3 studies [13, 41, 42] that were not included in the previous meta-analysis. Consistently with Kim et al., pooled data showed a significant reduction in short-term mortality (RR = 0.88, 95% CI: 0.82–0.95) and a smaller but significant reduction in poor neurological outcome (RR = 0.95, 95% CI: 0.90–0.99) in patients treated with TTM. Again, the beneficial effects of TTM on long-term survival and neurological recovery were still inconclusive since the 3 included studies did not report long-term outcomes.

In the current study, literature search resulted in 8 new additional OCSs (1618 cases and 2003 controls) but did not have additional RCTs investigating the role of TTM in nonshockable cardiac arrests. Compared with the 15 OCSs that were involved in previous meta-analysis [11, 45], the proportion of studies designed to specifically investigate the association between TTM and prognosis of nonshockable rhythms was increased (4/8 versus 2/15). Moreover, the number of studies with substantial risks of bias due to small sample size (less than 100 cases for hypothermia) was decreased (3/8 versus 12/15) in the 8 updated OCSs. Among the 8 OCSs, 7 studies [14–17, 40, 43, 44] reported short-term (hospital discharge or 1 month) outcomes and 2 studies [12, 15] reported long-term (6 months or 1 year) outcomes. The updated meta-analysis, therefore, could provide a comprehensive and appraisal of the effectiveness of TTM for nonshockable cardiac arrests. Consistent with the 2 previous reviews, pooled data validated that TTM was associated with improved short-term survival and neurological recovery with high confidence. More importantly, the results suggested that TTM also significantly improved long-term survival and neurological outcomes, despite the small number of trials included in this meta-analysis.

Even though the average NOS score indicated that all of the OCSs included in this review are of high quality, the quality of evidence for TTM benefits was “very low” for short-term outcome and “low” for long-term outcome when assessed with the GRADE criteria. The following reasons may account for the relative lower quality of evidence. First, most of the studies (17/23) had substantial risks of bias because they were not specifically designed to evaluate the beneficial effects of TTM in nonshockable rhythms. The subgroup of patients with PEA/asystole was quite small in sample size and less than 20 cases of hypothermia were involved in half of these studies (9/17). None of the 9 trials individually showed a significant improvement with TTM compared with normothermic control, which was probably because of inadequate statistical power, therefore resulting in a high degree of imprecision. Second, because of the absence of randomization, differences in patient and arrest characteristics between TTM and control groups were common in these studies. The reported selection bias included sex, age, hypertension, incident location, duration of arrest, witnessed arrest, and time to ROSC [12, 13, 15, 16, 41, 42, 44]. These discrepancies can lead to biased estimates of the treatment effects when one or more of the characteristics for which there are differences are related to the outcomes being measured. Third, the effects of TTM may be confounded by different cooling methods and rates of rewarming. For example, surface, invasive, and combined cooling techniques were applied and the rewarming rates ranged from 0.25 to 0.5°C/hr in the reported studies.

Although OCSs suggested that TTM is associated with a survival and neuroprotective benefit for nonshockable cardiac arrests, no randomized trials have validated the efficacy of TTM in survivors presenting with nonshockable rhythms. An ongoing multicenter RCT study in France with blinded outcome assessment in which 584 subjects with successfully resuscitated nonshockable cardiac arrests were allocated at random to either TTM or normothermia may afford certainty of the actual benefit of TTM in this population [46].

The physiological effects of hypothermia are thought to be multifactorial, including suppression of free radicals, enzymes, excitotoxic and inflammatory reactions, preservation of the blood-brain barrier following the disruptive effects of ischemia-reperfusion, and reduction of cerebral oxygen consumption and energy metabolism [47]. Compared to dysrhythmic arrest of cardiac origin, potential causes of PEA/asystole include degeneration of a primary shockable rhythm, respiratory distress, drowning, hypovolemia, acidosis, hyper/hypokalemia, hypothermia, drug overdose, cardiac tamponade, tension pneumothorax, and coronary or pulmonary artery thrombosis [5, 48, 49]. As patients with nonshockable initial rhythms differ in pathophysiology and are associated with significantly worse short- and long-term outcomes compared to those with shockable ones with or without hypothermia [17, 18, 21, 50], identification of particular subgroups of victims who may not benefit from TTM and development of alternative approaches are an unmet medical need in ameliorating the prognosis of these patients.

Although TTM benefits patients resuscitated from nonshockable cardiac arrests, its side effects should not be ignored. The available evidence in the included 2 studies suggests an association between TTM and the risk of infectious complications, which is consistent with a previous meta-analysis that risk of pneumonia and sepsis was increased in patients treated with hypothermia [51]. Clinicians, therefore, should cautiously assess patient's risk-benefit profile during TTM.

There are several limitations of the current study. First, the overall quality of the evidence was limited due to the small number of RCTs and small sample size of OCSs. However, the problem of small OCS sample size was likely ameliorated by their large number. Second, there were considerable differences in patient and arrest characteristics, together with cooling methodology among the OCSs included in this study. But the quantitative analysis of these studies was supported by the lack of statistical heterogeneity.

## 5. Conclusions

The present study suggests that TTM is associated with improved short- and long-term outcomes for adult patients resuscitated from nonshockable cardiac arrests. At the same time, incidence of infectious complications is increased for patients treated with hypothermia.

## Supplementary Material

The supplemental materials include search strategy used for the databases (Medline, EMBASE, and Cochrane databases), components of risk of bias for the individual randomized controlled trials, risk of bias assessment of the eligible observational cohort studies and GARDE profile for assessing quality of evidence.

## Figures and Tables

**Table 1:** Characteristics of included studies in the meta-analysis.

Author (year)	Study design	Country	Setting	Arrest location	Cooling methods	Nonshockable		Length of follow-up
						Total	Cooled	
Hachimi-Idrissi et al. (2005) [26]	RCT	Belgium	Single hospital	OHCA	External	33	16	6 months
Laurent et al. (2005) [27]	RCT	France	Single hospital	OHCA	Mixed	11	6	6 months
Holzer et al. (2006) [28]	OCS	Austria	Single hospital	Mixed	Internal	534	28	30 days
Oddo et al. (2006) [29]	OCS	Switzerland	Single hospital	OHCA	External	23	12	Discharge
Arrich et al. (2007) [30]	OCS	Europe	Multicenter	Mixed	Mixed	197	124	Discharge
Sunde et al. (2007) [31]	OCS	Norway	Single hospital	OHCA	Mixed	15	6	Discharge
Heer (2007) [32]	OCS	Germany	Single hospital	OHCA	Internal	18	10	Discharge
Rittenberger et al. (2008) [33]	OCS	USA	Single hospital	Mixed	Mixed	81	42	Discharge
Storm et al. (2008) [34]	OCS	Germany	Single hospital	OHCA	Mixed	49	18	Discharge
Bro-Jeppesen et al. (2009) [35]	OCS	Denmark	EMS + single hospital	OHCA	Mixed	34	13	Discharge/6 months
Gaieski et al. (2009) [36]	OCS	USA	Single hospital	OHCA	Mixed	18	9	Discharge
Whitfield et al. (2009) [37]	OCS	Australia	EMS + single hospital	OHCA	Mixed	28	15	Discharge
Don et al. (2009) [38]	OCS	USA	Single hospital	OHCA	External	313	122	Discharge
Derwall et al. (2009) [39]	OCS	Germany	EMS + single hospital	OHCA	Mixed	28	13	14 days
Testori et al. (2011) [12]	OCS	Austria	Single hospital	OHCA	Mixed	374	135	6 months
Dumas et al. (2011) [13]	OCS	France	Single hospital	OHCA	External	437	261	Discharge
Pfeifer et al. (2011) [40]	OCS	Germany	Single hospital	Mixed	Mixed	110	71	1 month
Storm et al. (2012) [41]	OCS	Germany	Single hospital	Mixed	Mixed	175	87	Discharge
Lundbye et al. (2012) [42]	OCS	USA	Single hospital	Mixed	Internal	100	52	ICU Discharge
Lindner et al. (2013) [14]	OCS	Norway	Single hospital	OHCA	Mixed	263	143	Discharge
Kozinski et al. (2013) [43]	OCS	Poland	Single hospital	OHCA	Internal	13	6	Discharge
Vaahersalo et al. (2013) [15]	OCS	Finland	Single hospital	OHCA	Mixed	223	70	Discharge/1 year
Perman et al. (2015) [16]	OCS	USA	Single hospital	Mixed	Unknown	519	262	Discharge
Doshi et al. (2016) [44]	OCS	USA	Multicenter	OHCA	Unknown	696	335	Discharge
Sung et al. (2016) [17]	OCS	USA	Single hospital	OHCA	Unknown	1423	596	Discharge

RCT: randomized controlled trials; OCS: observational cohort studies; OHCA: out-of-hospital cardiac arrest; EMS: emergency medical services.
