# Supplementary material for: The Role of Targeted Temperature Management in Adult Patients Resuscitated from Nonshockable Cardiac Arrests: An Updated Systematic Review and Meta-Analysis
Source: Biomed Res Int. 2016 Oct 25;2016:2350974. doi: 10.1155/2016/2350974 (PMC5099489; doi:10.1155/2016/2350974)
Supplement: Supplementary file 1 — The supplemental materials include search strategy used for the databases (Medline, EMBASE, and Cochrane databases), components of risk of bias for the individual randomized controlled trials, risk of bias assessment of the eligible observational cohort studies and GARDE profile for assessing quality of evidence. [file 2350974.f1.docx]

**Supplemental Table 1. Search strategy used for the databases (Medline, EMBASE, and Cochrane databases)**

| 1. exp Hypothermia/ or exp Hypothermia, Induced/  2. cooling. ab,ti.  3. chilling. ab,ti.  4. exp Body Temperature/  5. target temperature. ab,ti.  6. therapeutic hypothermia. ab,ti.  7. resuscitative hypothermia. ab,ti.  8. temperature control. ab,ti.  9. temperature management. ab,ti.  10. rewarming. ab,ti. or exp Rewarming/  11. chill therapy. ab,ti.  12. cooled. ab,ti.  13. mode of cooling. ab,ti.  14. external cooling. ab,ti.  15. endovascular cooling. ab,ti.  16. exp Body Temperature/ or normothermia. ab,ti.  17. cardiac arrest. ab,ti. or exp Heart Arrest/  18. exp Resuscitation/ or resuscitation.ab,ti. or exp  Cardiopulmonary Resuscitation/  19. reanimation. ab,ti.  20. exp Out-of-Hospital Cardiac Arrest/ | 21. heart stop. ab,ti.  22. return of spontaneous circulation. ab,ti.  23. pulseless ventricular tachycardia. ab,ti.  24. pulseless electrical activity. ab,ti.  25. asystole. ab,ti.  26. non-VF/VT. ab,ti.  27. non shockable rhythm. ab,ti.  28.neurological function. ab,ti.  29. neurological recovery. ab,ti.  30. neurological outcome . ab,ti.  31.cerebral performance category . ab,ti.  32. survival. ab,ti.  33. mortality . ab,ti.  34. exp Human/  35.1 or 2 or 3 or 4 or 5 or 6 or 7 or 8 or 9 or 10 or 11 or 12 or 13 or 14 or 15 or 16  36. 17 or or 18 or 19 or 20 or 21 or 22  37. 23 or 24 or 25 or 26 or 27  38. 28 or 29 or 30 or 31 or 32 or 33  39. 34 and 35 and 36 and 37 and 38 |
| --- | --- |

**Supplemental Table 2. Components of risk of bias for the individual randomized controlled trials**

| study | a1 | a2 | a3 | a4 | a5 | a6 | a7 | a8 |
| --- | --- | --- | --- | --- | --- | --- | --- | --- |
| Hachimi-Idrissi et al. (2005) | L | U | U | U | L | U | L | L |
| Laurent et al. (2005) | L | L | U | U | L | U | L | L |

a1: Sequence generation; a2: Allocation concealment; a3: Blinding; a4: Incomplete date outcomes; a5: Selective outcome reporting; a6: Baseline imbalance; a7: Source of funding bias; a8: Academic bias; H: High risk of bias; L: Low risk of bias; U: Uncertain risk of bias.

**Supplemental Table 3. Risk of bias assessment of the eligible observational cohort studies**

| Study (year) | selection | | | | comparability | outcome | | | Total score |
| --- | --- | --- | --- | --- | --- | --- | --- | --- | --- |
|  | A1 | A2 | A3 | A4 |  | B1 | B2 | B3 |  |
| Holzer et al. (2006) | * | * | * | * | 0 | * | * | * | 7 |
| Oddo et al. (2006) | * | * | * | * | ** | * | * | * | 9 |
| Arrich et al. (2007) | * | * | * | * | 0 | * | * | * | 7 |
| Sunde et al. (2007) | * | * | * | * | 0 | * | * | * | 7 |
| Heer (2007) | * | * | * | * | 0 | * | * | * | 7 |
| Rittenberger et al. (2008) | * | * | * | * | 0 | * | * | * | 7 |
| Storm et al. (2008) | * | * | * | * | 0 | * | * | * | 7 |
| Bro-Jeppesen et al. (2009) | * | * | * | * | 0 | * | * | * | 7 |
| Gaieski et al. (2009) | * | * | * | * | 0 | * | * | * | 7 |
| Whitfield et al. (2009) | * | * | * | * | ** | * | * | * | 9 |
| Don et al. (2009) | * | * | * | * | 0 | * | * | * | 7 |
| Derwall et al. (2009) | * | * | * | * | 0 | * | * | * | 7 |
| Testori et al. (2011) | * | * | * | * | * | * | * | * | 8 |
| Dumas et al. (2011) | * | * | * | * | * | * | * | * | 8 |
| Storm et al. (2012) | * | * | * | * | * | * | * | * | 8 |
| Pfeifer et al. (2011) | * | * | * | * | 0 | * | * | * | 7 |
| Lundbye et al. (2012) | * | * | * | * | * | * | * | * | 8 |
| Lindner et al. (2013) | * | * | * | * | 0 | * | * | * | 7 |
| Vaahersalo et al. (2013) | * | * | * | * | * | * | * | * | 8 |
| Kozinski et al. (2013) | * | * | * | * | 0 | * | * | * | 7 |
| Perman et al. (2015) | * | * | * | * | * | * | * | * | 8 |
| Doshi et al. (2016) | * | * | * | * | * | * | * | * | 8 |
| Sung et al. (2016) | * | * | * | * | ** | * | * | * | 9 |

A1: Representativeness of the exposed cohort; A2: Selection of the non-exposed cohort; A3: Ascertainment of exposure; A4: Demonstration that outcome of interest was not present at start of study; B1: Assessment of outcome; B2: Was follow-up long enough for outcomes to occur; B3: Adequacy of follow up of cohorts.

**Supplemental Table 4. GARDE profile for assessing quality of evidence**

| Quality assessment Summary of findings | | | | | | | | | | | | Importance |
| --- | --- | --- | --- | --- | --- | --- | --- | --- | --- | --- | --- | --- |
| No.of  studies | C1 | C2 | C3 | C4 | C5 | C6 | No.of patients | | Effect | | Quality |  |
|  |  |  |  |  |  |  | Therapeutic  hypothermia | Control | Relative(95%CI) | Absolute |  |  |
| Short-term survival | | | | | | |  | |  | |  |  |
| 19 | OCS | Serious^1^ | Serious^2^ | Serious^3^ | No serious imprecision | None | 596/2012  (29.6%) | 672/2802  (24.0%) | 1.42  [1.28,1.57] | 101 more per 1000(from 67 more to 137 more) | ⊕000  Very low | CRITICAL |
| Short-term neurological outcome | | | | | | |  | |  | |  |  |
| 17 | OCS | Serious^1^ | Serious^2^ | Serious^3^ | No serious imprecision | None | 320/1796  (17.8%) | 317/2420  (13.1%) | 1.63  [1.39,1.91] | 83 more per 1000(from 51 more to 119 more) | ⊕000  Very low | CRITICAL |
| Long-term survival | | | | | | |  | |  | |  |  |
| 2 | RCT | Serious^1^ | No serious inconsistency | Serious^4^ | Serious^5^ | None | 5/22  (22.7%) | 2/22  (9.1%) | 2.22  [0.56,8.85] | 111 more per 1000(from 40 more to 714 more) | ⊕⊕00  Low | CRITICAL |
| Long -term neurological outcome | | | | | | |  | |  | |  |  |
| 1 | RCT | Serious^6^ | No serious inconsistency | Serious^7^ | Serious^5^ | None | 2/16  (12.5%) | 0/17  (0.0%) | 5.29  [0.27,102.49] | -------- | ⊕⊕⊕0  Moderate | CRITICAL |
| Long -term survival | | | | | | |  | |  | |  |  |
| 3 | OCS | Serious^1^ | No serious inconsistency | No serious | No serious imprecision | None | 79/218  (36.2%) | 90/413  (21.8%) | 1.64  [1.27,2.12] | 139more per 1000(from 59 more to 244 more) | ⊕⊕00  Low | CRITICAL |
| Long -term neurological outcome | | | | | | |  | |  | |  |  |
| 2 | OCS | Serious^1^ | No serious inconsistency | No serious | No serious imprecision | None | 60/205  (29.3%) | 79/392  (20.2%) | 1.42  [1.07,1.90] | 85 more per 1000(from 14 more to 181 more) | ⊕⊕00  Low | CRITICAL |
| Infectious complication | | | | | | |  |  |  |  |  |  |
| 2 | OCS | Serious^1^ | No serious inconsistency | No serious | No serious imprecision | None | 205/331  61.9%) | 152/329  (46.2%) | 1.46  [1.26,1.70] | 213 more per 1000(from 120 more to 323more) | ⊕000  Very low | CRITICAL |

C1:Design; C2:Limitations; C3:Inconsistency;C4: Indirectness; C5:Imprecision; C6:Other considerations.

^1^ All studies had substantial risks of bias.

^2^ Significant heterogeneity exists in the meta-analysis for short term survival in OCS studies.

^3^ The eleven studies did not report comparability of cohorts.

^4^ One trial did not report the number of the screened patients. The other trial included only 17% of the screened patients with ROSC and only a subset (asystole) of the target cardiac arrest population.

^5^ The sample size of the trial is limited. The 95% CI for the overall RR showed a wide variety.

^6^ The trial had substantial risk of bias.

^7^ The trial did not report the number of the screened patients.
